# Immune Disorders in Hashimoto's Thyroiditis: What Do We Know So Far?

**DOI:** 10.1155/2015/979167

**Published:** 2015-04-27

**Authors:** Aleksandra Pyzik, Ewelina Grywalska, Beata Matyjaszek-Matuszek, Jacek Roliński

**Affiliations:** ^1^Department of Endocrinology, Medical University, 20-059 Lublin, Poland; ^2^Department of Immunology, Medical University, 20-059 Lublin, Poland

## Abstract

This review of literature attempts to identify the factors that are involved in the pathogenesis of Hashimoto thyroiditis, an immune defect in an individual with genetic susceptibility accompanied with environmental factors. The frequency of Hashimoto's disease is a growing trend and among Caucasians it is estimated at approximately 5%. The dysfunction of the gland may be clinically evident (0.1–2% of the population) or subclinical (10–15%). The pathology is diagnosed five to ten times more often in women than men and its incidence increases with the age (the peak of the number of cases is between 45 and 65); however, it can also be diagnosed in children. The pathogenesis of Hashimoto's thyroiditis is still not fully comprehended. In the etiology of Hashimoto thyroiditis excessively stimulated T CD4+ cells are known to play the most important role. Recent research has demonstrated an increasing role of newly discovered cells such as Th17 (CD4+IL-17+) or T regulatory cells (CD4+CD25+^high^FoxP3+) in the induction of autoimmune disorders. The process of programmed cell death also plays an equally important role in the pathogenesis and the development of hypothyroidism.

## 1. Introduction 

Autoimmune Thyroid Diseases (AITD) constitute 30% of all the autoaggressive diseases and are qualified as organ-specific out of which Hashimoto's thyroiditis (HT; chronic lymphocytic thyroiditis) and Graves' disease are the most crucial [1]. HT was described in 1912 by Hakaru Hashimoto but not until the 1950s had its autoimmune aspect been demonstrated. Recent years have shown that HT development depends on an immune defect in an individual with genetic susceptibility together with environmental factors [2]; however, the pathogenesis of Hashimoto's thyroiditis is still not fully comprehended. Morphologically, the disease consists in a gradual atrophy of thyroid tissue following gland invasion with lymphocytic cells, follicular atrophy, and hyperemia accompanied by oncocytic metaplasia of follicular cells [3]. This leads to the development of hypothyroidism, though the disease may occur with a normal thyroid activity. The dysfunction of the gland may be clinically evident (0.1–2% of the population) or subclinical (10–15%) which depends on the scope of the damaged thyroid parenchyma [4]. On the basis of current data there is no clear statement about the correlation between antibody titers and the risk of (subclinical) hypothyroidism. Bossowski and Otto-Buczkowska stated that serologically antithyroid antibodies, especially against thyroperoxidase (anti-TPO) and antithyroglobulin (anti-Tg) and more rarely TSH-stimulation blocking antibody (TSBAb), correlate positively with an increased inflammatory reaction in the thyroid and with the development of hypothyroidism [5]. Garber et al. claimed that the presence of elevated anti-TPO titers in patients with subclinical hypothyroidism helped to predict progression to overt hypothyroidism—4.3% per year with anti-TPO versus 2.6% per year without elevated anti-TPO titers [6]. According to Schott and Scherbaum an increased TSH level and clinical hypothyroidism were significantly associated with TPO antibodies, but not with Tg antibodies [7]. Yoshida et al. showed a highly significant correlation between morphological and serological findings in a postmortem examination. In their study, the incidence of a small thyroid gland of less than 15 g in weight was higher in patients with lymphocytic infiltration and/or positive autoantibodies than in patients with a normal thyroid gland [8]. On the other hand, studies conducted by Peretianu showed that there was no correlation between the anti-TPO level, echographic pattern and thyroid function, but there was a highly correlative relationship between the echographic pattern and anti-TPO levels in Hashimoto patients [9]. The frequency of Hashimoto's disease among Caucasians is estimated at approximately 5%. The pathology is diagnosed five to ten times more often in women than men and its incidence increases with the age (the peak is between 45 and 65). It is significantly more frequent in individuals who suffer from other autoaggressive diseases, for example, Addison's disease, diabetes mellitus type 1 (T1DM), rheumatoid arthritis, or systemic lupus erythematosus (SLE), which are classified as autoimmune polyendocrine syndromes [5].

## 2. Antithyroid Antibodies

Already in 1990 it was proven that using Tg or TPO as antigens induced experimental autoimmune thyroiditis in mice, which suggested that these antigens might play a role in the pathogenesis of HT in humans [10, 11]. Antithyroid antibodies have an ability to fix complement if they are of an appropriate IgG subclass. As a result, complement-dependent antibody-mediated cytotoxicity leads to more damage of thyroid tissue in comparison to T cells and cytokine-mediated apoptosis [12]. More important in presenting thyroid antigens to the T cells is the role of thyroid antibody secreting B cells. The thyroglobulin gene may be a susceptibility gene for autoimmune thyroiditis coding for thyroglobulin variants of different immunogenicity [13].

## 3. B Lymphocytes

B cells from thyroid tissue of patients with HT are activated as indicated by their ability to secrete antithyroid antibodies spontaneously* in vitro*. Thus, the thyroid may be a major site for thyroid antibody secretion as it is evidenced in the decline in serum of thyroid antibody concentrations that occurs after surgery and during administration of antithyroid drugs to patients with this disorder [14]. However, there is also evidence that extrathyroidal lymphoid tissues may contribute to antibody production [15].

In their study Beń-Skowronek et al. [16] found an increased number of plasma cells in HT. CD79 alpha+ B cells constituted almost half of the cells in the mononuclear lymphatic infiltrates; in HT, foci of destruction of thyroid follicles and thyrocytes were visible at the sites of accumulation of plasma cells.

Kawashima et al. [17] screened serum total IgG levels in the patients with HT to illustrate the prevalence of IgG4-related thyroiditis. The levels of IgG and IgG4 positively correlated with the titers of anti-Tg antibody or anti-TPO antibody. This observation suggests that at least a small number of patients with HT with high titers of antithyroid antibodies may also suffer from the IgG4-related thyroiditis.

## 4. T Lymphocytes

**Figure 1:**
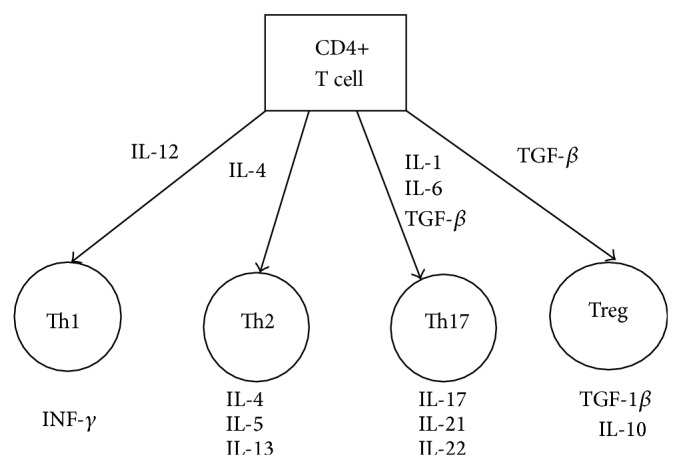
T CD4+ cell differentiation.

Excessively stimulated T cells CD4+ are known to play the main role in the pathogenesis of HT (Figure 1). T cells perform two functions in the pathogenesis of HT. T helper type 2 Th2 cells lead to an excessive stimulation and production of B cells and plasmatic cells which produce antibodies against thyroid antigens leading to thyroiditis [18]. T helper type 1 (Th1) and Th2 cells produce interferon- (IFN-) gamma, and interleukin- (IL-) 4, respectively. Nanba et al. reported that IFN-gamma and IL-4 gene polymorphisms, which are related to higher IFN-gamma and lower IL-4 production, respectively, are more frequent in patients with severe HT than in those with mild HT [19]. Th1 cells activate cytotoxic lymphocytes and macrophages, which directly affect thyroid tissue by destroying thyroid follicular cells. In the tissues of the thyroid in patients with HT Th1 are the predominant cells. Histopathological studies have shown that more T cells have been observed in HT both in the parenchyma and in the lymphatic infiltrations. In HT, damaged thyroid follicles with apoptotic thyrocytes (pyknotic nuclei, condensed cytoplasm with enlarged mitochondria and endoplasmic reticulum cisterns) were visible in this area. A number of CD4+ T cells in the thyroid infiltrates in HT were significantly decreased in the interstitium. Observations under a light microscope revealed that T suppressor/cytotoxic cells were accumulated at the sites of destruction of thyroid follicles. These sites were surrounded by connective tissue fibers and fibroblasts [16].

Recent research has demonstrated a prominent role of newly discovered cells such as Th17 (CD4+IL-17+) or Treg lymphocytes (CD4+CD25+^high^FoxP3+) in the induction of autoimmune disorders [20]. Zha et al. [21] observed a significant infiltration of lymphatic cells in the thyroid specimens of HT, and they could not find lymphatic invasion in all the normal thyroid tissues. The authors revealed that thyroid tissue in Hashimoto's disease was mainly infiltrated with B cells CD20+. Immunohistochemical analysis showed a meaningful infiltration of lymphatic cells in the thyroid specimens from all HT patients, while no clear infiltration of lymphatic cells in the normal thyroid specimens was found. In the majority of the patients an infiltration with T cells with CD4+ and CD8+ expression was found and in nine out of 17 patients FoxP3+ expression was observed. In the study conducted in 2007, Beń-Skowronek et al. [22] found a significant difference in the profile analysis of lymphocyte subsets in Hashimoto's disease and the control group. The thyroid preparations were observed in the patients with HT forming a large number of massive infiltrations of lymphocytes and numerous lymphoid follicles. The most abundant were the B lymphocytes, which accounted for over 10% of the interstitial tissue and 51% of cells with lymphocyte infiltration. In contrast, virtually no CD4+ Th cells were observed. There was also a large percentage of CD8+ suppressor cells. In the thyroid tissue of the control group there was a small number of lymphocytes, and most numerous were the CD79+ B cells. In the study of Cunha et al. the authors demonstrated no normal thyroid cases with FoxP3+ lymphocytes [23].

## 5. T Helper 17 Cells (Th17)

Th17 lymphocytes serve as a pathogenic factor in the development of various diseases. These could be autoimmune diseases like psoriasis, multiple sclerosis, rheumatoid arthritis or inflammatory bowel disease or neoplasms, allergies, engraftment, or transplant rejection. However, their role in AITD is still debatable. Th17 cells account for approximately 1% of CD4+ lymphocytes in blood serum and take part in the immune response against intercellular antigens. They are characterized by expression markers such as CCR6 (CD196), IL-23R, IL-12R-beta2, CD49, and CD161 and produce proinflammatory cytokines mainly: IL-17A, IL-17F, IL-21, IL-9, IL-22, and TNFA. They develop from T helper cells under the influence of various factors of differentiation, growth, and stabilization such as TGFB plus IL-6, IL-21, and IL-23 and transcription factors like STAT3, RORgammat, and RORalpha [24–26]. Liu et al. and Qin et al. found that HT patients had a significantly increased serum concentration of IL-6 and IL-23 in comparison with healthy controls [27, 28], whereas Kimura and Kishimoto showed that IL-6 induces Th17 differentiation together with TGFB [29]. This T cell differentiation is presented in Figure 1. Bossowski et al. [30] demonstrated an elevated level of Th17 cells in children with untreated Hashimoto's disease, which suggests the participation of these cells in the induction and development of the disease. However, they did not demonstrate such a relation in Graves' disease. Similarly, in the research by Li et al. [31] a significantly higher concentration of IL-17 was visible in HT compared to in the thyroid cancer, in the nodular goiter, or in the studied group. Further research by Li et al. suggested a negative relationship between the level of IL-17 and the stage of hypothyroidism among patients with Hashimoto's disease. Histopathological examinations have shown a strong relationship between the concentration of IL-17 and the stromal fibrosis in the gland, which points to the fact that the presence of IL-17 increases local inflammation and leads to the fibrosis and atrophy of thyrocytes. Additionally, it was concluded that the impact of sodium iodide concentrations on the development of Th17 and Th1 lymphocytes, which can serve as inhibitors of regulatory T cells, might be various, whereas the research by Shi et al. [32] demonstrated that mRNAs of IL17 and transcription factor were significantly increased in PBMC (peripheral blood mononuclear cell) from patients with HT. Wang et al. tried to answer the question of why the number of Th17 rises in HT. The authors observed an elevated concentration of proinflammatory leptin in blood of the patients and concluded that this cytokine could induce the proliferation of T lymphocytes and promote immune response in the direction of Th17 [33].

## 6. Regulatory T (Treg) Cells 

Regulatory T cells are T helper cells CD4+ which additionally demonstrate an expression of a reactor for IL-2 alpha chain (CD25) and are responsible for suppressing autoimmunization. They constitute only 5–10% of Th lymphocytes. It has been shown that they not only are able to suppress the proliferation and produce cytokines by T cells CD4+CD25, but also suppress proliferation of CD8+ T lymphocytes* in vitro*, NK cells or dendritic cells [34–37]. Moreover, today we know that inhibition of follicular T helper cells by CD8+ regulatory T cells is essential for self-tolerance [38]. In 1995 Sakaguchi et al. [39] demonstrated that depriving mice of transgenic T cells CD25+ led to the occurrence of organ-specific autoaggressive diseases and they unambiguously confirmed the participation of these cells in suppressing the process of autoaggression. Two subpopulations of these cells were discovered: the so-called natural regulatory T cells and induced regulatory cells, which are produced on the boundary of CD4+CD25− (iTreg; Tr1, Th3 lymphocytes, and Tr1-like cells). Natural Treg cells CD4+25+ are produced in the thymus wherein the course of maturation becomes resistant to apoptosis. Some morphological changes take place on the surface of the cell and they acquire specific nuclear transcription factor FoxP3 which is responsible for suppressing an excessive reaction of the immune system. FoxP3 codes scurfin, that is, the negative transcription factor which blocks the expression of proinflammatory cytokines that activate Th1 lymphocytes [40, 41]. The other cell group T CD4+ which has a regulatory function creates cells that are produced outside the thymus, that is, on the boundary of lymphocytes T CD4+ CD25−, mainly Tr1. They are also antigen specific; they synthesize IL-10 and TGFB and can, but do not have to, demonstrate CTLA-4, CD25, GITR, or FoxP3 expression [42, 43]. The research* in vitro *and* in vivo* conducted in recent years confirmed a regulatory function of CD4+CD25+ lymphocytes, their key role in the immune response, and their participation in the development of autoimmune diseases of the thyroid [34]. Marazuela et al. [42] discovered that in the patients with AITD there are more T CD4+ cells which demonstrate a disturbed expression of IL-10, TGFB, genes for transcription factors FoxP3, STAT1, and STAT3, and critical genes for Treg cells (such as encoding OX40, 4-1BB, ICOS, GITR, and CTLA-4). There is some evidence supporting the association between the CTLA4 locus and Hashimoto thyroiditis [44]. The research by Nakano et al. [45] presented a decreased level of regulatory T cells among intrathyroid lymphocytes in individuals with autoimmune thyroid dysfunction in comparison to the control group while naturally present Treg underwent apoptosis. This was confirmed in the research by Bossowski et al. [2] conducted among children with a newly diagnosed AIDT. Further research from 2013 showed an increasing percentage of these lymphocytes in the patients who underwent L-thyroxine replacement therapy. A negative correlation was observed between the percentage of Treg CD4+CD25+ high lymphocytes and the concentration of anti-TPO antibodies in individuals who had not been treated [41], unlike in the case of the research by Glick et al. [46] who did not find the difference in the percentage of regulatory T cells in CD4+ population in a group of patients suffering from autoimmune diseases of the thyroid. However, Glick et al. noticed that regulatory T cells in patients with thyroid dysfunction had a lower ability to suppress the proliferation of the rest of lymphocytes [46]. Zha et al. [21] raised a similar concern. They found that regulatory T cells could be damaged and they do not fulfill their function. Additionally, damaged Treg can destroy immune tolerance and induce autoimmunization, which Marazuela et al. [42] confirmed in their research. The results show that in the treatment of Hashimoto's disease increasing the number of regulatory lymphocytes can be more effective if it is accompanied with the activation of their function, for example, by vitamin D3 [47].

## 7. Apoptosis

**Figure 2:**
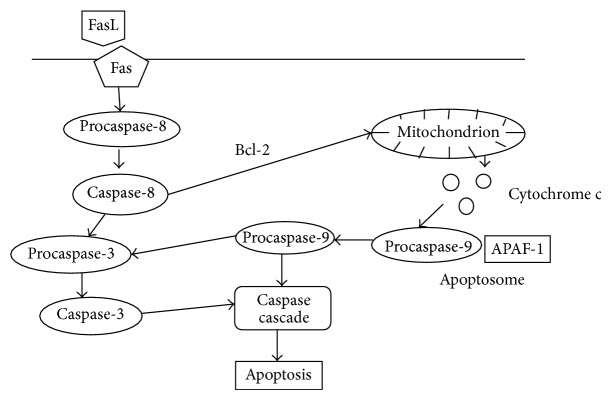
Schematic representation of apoptosis (extrinsic and intrinsic pathways) in HT.

A programmed cell death is a physiological process necessary in an organism in order to retain homeostasis. It aims at removing excessive redundant or autoreactive cells in the process of peripheral deletion. In HT, apoptosis plays an important role because of the extrinsic pathway of apoptosis induction by means of binding FasL (CD95L) with its receptor Fas (CD95) (Figure 2). After binding the ligand, Fas combines with adaptive protein, for example, FADD (Fas-associated death domain protein), which binds procaspase-8, that is, a family of protein cysteine proteases also described as FLICE, FADD-like interleukin 1b-converting enzyme, creating DISC (death-induced signaling complex). Activated caspase-8 induces cysteine proteases or caspase-3 complex which selectively affects proteins, suppresses their activity or activates them, and leads to the final destruction of a cell. Moreover, components activated in this process, caspase-8 molecules, by means of proapoptotic protein from Bcl-2 family (B cell lymphoma-2), Bid molecule (BH3 interacting domain death agonist), initiate intrinsic pathway of the activation of apoptosis releasing cytochrome c. Cytochrome c with procaspase-9 and apoptotic protease activating factor-1 (APAF-1) create a complex—apoptosome. Activated caspase-9 reactivates caspase-3, which leads to the disintegration of the nucleus and cytoplasmic substrates. All these processes lead to cell death. Intrinsic pathway (mitochondrial) can be also activated by a direct damage of the essential cellular structures such as DNA, metabolic misalignment, or the disruption of the cell cycle [48, 49]. In tests on animal models and people it has been demonstrated that as a result of hereditary Fas or FasL gene mutation, an accumulation of T cells and the development of lymphadenopathy or autoimmune diseases, such as HT, are present. Bossowski et al. [48] described a significantly higher percentage of the expression of apoptotic molecules Fas/FasL on the surface of thyroid cells and a significant expression of proapoptotic proteins Fas/FasL in thyroid cells. Additionally, they observed a significantly lower percentage of lymphocytes incoming to the thyroid gland which demonstrated the expression of these molecules. This leads to the destruction of follicular cells of the thyroid either in the mechanism of apoptosis or as a result of the activity of T cells and finally results in the development of hypothyroidism. Similar conclusions were drawn by Giordano et al. [50]. Later, Stassi et al. [51] observed that Fas and FasL expression are elevated in thyrocytes in an active phase of HT, but according to the researchers the proteins also underlie the expression in normal thyrocytes. Nonetheless, their concentration is insufficient to initiate apoptosis. Moreover, they stated that the changes in the distribution of apoptotic markers on thyrocytes may be caused by proinflammatory cytokines released by macrophages and Th1 lymphocytes such as IFNG, TNFA, and IL-12. Among others, they are responsible for increased Fas expression on the surface of thyrocytes and elevated concentration of procaspases 8, 10, and 7 and increased Bid expression, Bcl-xL, and Bak to a lesser extent as well as a slightly decreased Bcl-2 concentration. This stimulation causes an activation of apoptosis in the mechanism of suicide or fratricide leading to the destruction of follicular cells of the thyroid, which Su et al. [52] confirmed in another research. There are many known apoptotic inhibitors, that is, FAP-1 (Fas-associated phosphatase 1), FLIP (FLICE-like inhibitory protein), Bcl-2 protein family (Bcl-2 and Bcl-xL), and IAP (inhibitory apoptotic proteins). Disturbance in their expressions in thyrocytes plays an important role in the pathogenesis and destruction of thyroid parenchyma in HT [48]. The conducted research demonstrated a decreased level of Bcl2 in patients suffering from HT as compared to the control group, while in lymphocytes which infiltrate the thyroid the level was high. This stimulates apoptosis of thyrocytes and damage of thyroid parenchyma [49]. It was shown that apoptotic factor of thyrocytes correlates with clinical symptoms of AITD [53–55]. We also know that TSH, TSAb (thyroid stimulating antibody), and TSBAb (thyroid stimulating blocking antibody) inhibit apoptosis by means of decreasing Fas expression [49, 56, 57]. In contrast, Bona et al. [58] observed a direct correlation between the concentration of anti-TPO antibodies in blood serum and the resistance of T cell to Fas-induced apoptosis in a group of untreated patients. A similar relationship was not observed in the group of patients who received a replacement therapy. Another research by Feldkamp et al. [56, 57] studied the influence of thyroid hormones (T3, T4) on the intensification of apoptosis among T lymphocytes and the decreased expression of intracellular antiapoptotic Bcl-2. The activity of apoptosis also depends on the iodine concentration. Low levels inhibit while high induce a programmed cell death which is dependent on Fas [49, 56, 57]. It is worth mentioning that soluble forms of Fas and FasL (sFas, sFasL) present in blood serum which are created in the process of alternative splicing prevent apoptosis by means of interference in Fas/FasL interaction. Patients with HT have a reduced level of these particles in blood serum [57].

## 8. TRAIL (TNF-Related Apoptosis-Inducing Ligand)

TNF-related apoptosis-inducing ligand also belongs to a TNF super-family and it is able to induce apoptosis in neoplastic cells. The increase of the expression of these receptors under the influence of certain proinflammatory cytokines such as IFNG or TNF was demonstrated in the research by Fang et al. [15]. They showed that TRAIL can also protect the thyroid against fibrosis in animal models. Mice which underwent the neutralization of endogenic TRAIL had an increased concentration of proinflammatory cytokines (IFNG, TNFA, IL-17, or TGFB) which take part in the process of fibrosis. They also had a decreased expression of anti-inflammatory cytokines such as IL-10 and IL-13, increased levels of FasL proteins and apoptotic molecules FLIP and Bcl-xL on inflammatory cells within the thyroid, and, at the same time, decreased levels of thyrocytes. Finally, programmed cell death was observed. Researchers suggested that TRAIL protein can belong to a subfamily of inhibitors of autoimmune diseases which inhibit inflammation.

## 9. Bystander Activation

The arrival of a thyroid-cell virus or activated nonspecific lymphocytes within the thyroid may cause local release of cytokines which may activate resident local thyroid-specific T cells. This bystander effect hypothesis has been supported by the studies on animal models of experimental autoimmune thyroiditis conducted by Arata et al. [59].

## 10. Thyroid-Cell Expression of HLA Antigens 

MHC class II molecules are present on thyroid follicular cells in patients with Hashimoto's thyroiditis but not in normal subjects. Expression of these molecules on thyroid follicular cells can be induced by interferon gamma and other products of T cells when the T cells are activated (e.g., by a viral infection) [60]; the expression can also be induced directly by viruses [61, 62]. Thyroid cells expressing MHC class II molecules are able to present antigens, either foreign or self, to T cells, thereby activating the T cells. The following observations provide an indirect support for a hypothesis that the induction of MHC class II molecules on thyroid follicular cells by interferon gamma can induce autoimmune thyroiditis in susceptible mice [63]. These findings strongly support the view that infection may induct the expression of MHC class II molecules on human thyroid cells and that these cells may act as antigen-presenting cells, thereby initiating a thyroid autoimmune response. Intrathyroidal dendritic cells and B cells may also serve as antigen-presenting cells and may provide important costimulatory molecules for effective antigen presentation.

## 11. Conclusions

In spite of various research conducted in recent years, the pathogenesis of Hashimoto's thyroiditis is still not fully comprehended. There is no doubt that Th1 lymphocytes participate in the development of this disease, but it seems that they do not play as an important role as it had been thought. Moreover, the significance of the participation of newly discovered subgroups of CD4+ such as Th17 in the etiopathogenesis of HT is still debatable. Many tests* in vitro* demonstrate an essential function of Treg in autoimmunological disorders. Various studies conducted on animal models show a strong correlation between the decrease in the number or impairment of the function of regulatory T cells and the development of thyroiditis. What is more, lymphocyte infusion from healthy mature mice prevented the development of these disorders. An increase of the level of regulatory T cells in the course of treatment with L-thyroxine was also observed. This may result from the suppression of differentiation of lymphocytes in the direction of Th1 by suppressing the production of cytokines participating in the maturation of this cell subclass such as IL-12 or INFG. It also seems that achieving euthyreosis by stopping the inflammation in the thyroid leads to the termination of autoaggression, which encourages the correct function of regulatory T cells. The process of apoptosis is also important in the pathogenesis and the development of hypothyroidism. The conducted research has shown a significantly higher level of the expression of apoptotic molecules and proapoptotic proteins on the surface of thyroid cells, which leads to the destruction of follicular cells of the thyroid in apoptosis. On the other hand, it has been shown that a significantly lower percentage of the lymphocytes incoming to the thyroid demonstrate the expression of apoptotic molecules. This results in the direct destruction of the thyroid parenchyma. The disorders in the expression of apoptotic inhibitory molecules in thyrocyte also play a significant role in the pathogenesis and destruction of the thyroid parenchyma in HT.

## References

[1] Brown R. S. (2009). Autoimmune thyroid disease: unlocking a complex puzzle. *Current Opinion in Pediatrics*.

[2] Bossowski A., Moniuszko M., Dąbrowska M. (2011). Analysis of T regulatory cells in the peripheral blood in children and adolescents with Graves' disease and Hashimoto's thyroiditis. *Endokrynologia Pediatryczna*.

[3] Pearce E. N., Farwell A. P., Braverman L. E. (2003). Thyroiditis. *The New England Journal of Medicine*.

[4] Socha K., Dziemianowicz M., Omeljaniuk W., Soroczyńska J., Borawska M. (2012). Dietary habits and the concentration of selenium in serum of patients with Hashimoto disease. *Problemy Higieny i Epidemiologii*.

[5] Bossowski A., Otto-Buczkowska E. (2007). Schorzenia tarczycy o podłożu autoimmunologicznym. *W: Pediatria —co nowego? Pod redakcją Ewy Otto-Buczkowskiej*.

[6] Garber J. R., Cobin R. H., Gharib H. (2012). Clinical practice guidelines for hypothyroidism in adults: cosponsored by the American Association of Clinical Endocrinologists and the American Thyroid Association. *Thyroid*.

[7] Schott M., Scherbaum W. A. (2006). Autoimmune Thyroid disease. *Deutsches Arzteblatt*.

[8] Yoshida H., Amino N., Yagawa K. (1978). Association of serum antithyroid antibodies with lymphocytic infiltration of the thyroid gland: studies of seventy autopsied cases. *The Journal of Clinical Endocrinology & Metabolism*.

[9] Peretianu D. (2005). Antithyroperoxidase antibodies (ATPO) in Hashimoto thyroiditis: variation of levels and correlation with echographic patterns. *Acta Endocrinology*.

[10] Kotani T., Umeki K., Hirai K., Ohtaki C. (1990). Experimental murine thyroiditis induced by porcine thyroid peroxidase and its transfer by the antigen-specific T cell line1000. *Clinical and Experimental Immunology*.

[11] Matsuoka N., Unger P., Ben-Nun A., Graves P., Davies T. F. (1994). Thyroglobulin-induced murine thyroiditis assessed by intrathyroidal T cell receptor sequencing. *Journal of Immunology*.

[12] Chiovato L., Bassi P., Santini F. (1993). Antibodies producing complement-mediated thyroid cytotoxicity in patients with atrophic or goitrous autoimmune thyroiditis. *The Journal of Clinical Endocrinology and Metabolism*.

[13] Ban Y., Greenberg D. A., Concepcion E., Skrabanek L., Villanueva R., Tomer Y. (2003). Amino acid substitutions in the thyroglobulin gene are associated with susceptibility to human and murine autoimmune thyroid disease. *Proceedings of the National Academy of Sciences of the United States of America*.

[14] McGregor A. M., Ibbertson H. K., Smith B. R., Hall R. (1980). Carbimazole and autoantibody synthesis in Hashimoto's thyroiditis. *British Medical Journal*.

[15] Fang Y., Sharp G. C., Yagita H., Braley-Mullen H. (2008). A critical role for TRAIL in resolution of granulomatous experimental autoimmune thyroiditis. *The Journal of Pathology*.

[16] Beń-Skowronek I., Szewczyk L., Kulik-Rechberger B., Korobowicz E. (2013). The differences in T and B cell subsets in thyroid of children with Graves' disease and Hashimoto's thyroiditis. *World Journal of Pediatrics*.

[17] Kawashima S. T., Tagami T., Nakao K. (2014). Serum levels of IgG and IgG4 in Hashimoto thyroiditis. *Endocrine*.

[18] Weetman A. P., McGregor A. M. (1994). Autoimmune thyroid disease: further developments in our understanding. *Endocrine Reviews*.

[19] Nanba T., Watanabe M., Inoue N., Iwatani Y. (2009). Increases of the Th1/Th2 cell ratio in severe Hashimoto's disease and in the proportion of Th17 cells in intractable Graves' disease. *Thyroid*.

[20] Korn T., Bettelli E., Oukka M., Kuchroo V. K. (2009). IL-17 and Th17 cells. *Annual Review of Immunology*.

[21] Zha B., Huang X., Lin J., Liu J., Hou Y., Wu G. (2014). Distribution of lymphocyte subpopulations in thyroid glands of human autoimmune thyroid disease. *Journal of Clinical Laboratory Analysis*.

[22] Beń-Skowronek I., Szewczyk L., Sierocińska-Sawa J., Korobowicz E. (2007). Subpopulacje limfocytów w tkance tarczycowej w autoimmunologicznych i nieautoimmunologicznych chorobach tarczycy u dzieci. *Endokrynologia Pediatryczna*.

[23] Cunha L. L., Morari E. C., Nonogaki S., Soares F. A., Vassallo J., Ward L. S. (2012). Foxp3 expression is associated with aggressiveness in differentiated thyroid carcinomas. *Clinics*.

[24] Miossec P., Kolls J. K. (2012). Targeting IL-17 and T_H_17 cells in chronic inflammation. *Nature Reviews Drug Discovery*.

[25] Wilke C. M., Bishop K., Fox D., Zou W. (2011). Deciphering the role of Th17 cells in human disease. *Trends in Immunology*.

[26] Zambrano-Zaragoza J. F., Romo-Martínez E. J., de Jesús Durán-Avelar M., García-Magallanes N., Vibanco-Pérez N. (2014). Th17 cells in autoimmune and infectious diseases. *International Journal of Inflammation*.

[27] Liu Y., Tang X., Tian J. (2014). Th17/Treg cells imbalance and GITRL profile in patients with hashimoto's thyroiditis. *International Journal of Molecular Sciences*.

[28] Qin Q., Liu P., Liu L. (2012). The increased but non-predominant expression of Th17- and Th1-specific cytokines in hashimoto's thyroiditis but not in graves' disease. *Brazilian Journal of Medical and Biological Research*.

[29] Kimura A., Kishimoto T. (2010). IL-6: regulator of Treg/Th17 balance. *European Journal of Immunology*.

[30] Bossowski A., Moniuszko M., Idźkowska E. (2012). Evaluation of CD4^+^CD161^+^CD196^+^ and CD4^+^IL-17^+^ Th17 cells in the peripheral blood of young patients with Hashimoto's thyroiditis and Graves' disease. *Pediatric Endocrinology, Diabetes, and Metabolism*.

[31] Li D., Cai W., Gu R. (2013). Th17 cell plays a role in the pathogenesis of Hashimoto's thyroiditis in patients. *Clinical Immunology*.

[32] Shi Y., Wang H., Su Z. (2010). Differentiation imbalance of Th1/Th17 in peripheral blood mononuclear cells might contribute to pathogenesis of Hashimoto's thyroiditis. *Scandinavian Journal of Immunology*.

[33] Wang S., Baidoo S. E., Liu Y. (2013). T cell-derived leptin contributes to increased frequency of T helper type 17 cells in female patients with Hashimoto's thyroiditis. *Clinical and Experimental Immunology*.

[34] Lewkowicz P., Lewkowicz N., Tchórzewski H. (2005). CD4^+^CD25^+^ T regulatory cells: their physiology and role in modulating immune response. *Postępy Higieny i Medycyny Doświadczalnej*.

[35] Misra N., Bayry J., Lacroix-Desmazes S., Kazatchkine M. D., Kaveri S. V. (2004). Cutting edge: human CD4^+^CD25^+^ T cells restrain the maturation and antigen-presenting function of dendritic cells. *Journal of Immunology*.

[36] Trzonkowski P., Szmit E., Myśliwska J., Dobyszuk A., Myśliwski A. (2004). CD4^+^CD25^+^ T regulatory cells inhibit cytotoxic activity of T CD8^+^ and NK lymphocytes in the direct cell-to-cell interaction. *Clinical Immunology*.

[37] Baecher-Allan C., Viglietta V., Hafler D. A. (2004). Human CD4+CD25+ regulatory T cells. *Seminars in Immunology*.

[38] Kim H. J., Verbinnen B., Tang X., Lu L., Cantor H. (2010). Inhibition of follicular T-helper cells by CD8^+^ regulatory T cells is essential for self tolerance. *Nature*.

[39] Sakaguchi S., Sakaguchi N., Asano M., Itoh M., Toda M. (1995). Immunologic self-tolerance maintained by activated T cells expressing IL-2 receptor alpha-chains (CD25). Breakdown of a single mechanism of self-tolerance causes various autoimmune diseases. *The Journal of Immunology*.

[40] Hori S., Nomura T., Sakaguchi S. (2003). Control of regulatory T cell development by the transcription factor Foxp3. *Science*.

[41] Bossowski A., Moniuszko M., Dabrowska M. (2013). Lower proportions of CD4+CD25^high^ and CD4 +FoxP3, but not CD4+CD25+CD127^low^ FoxP3^+^T cell levels in children with autoimmune thyroid diseases. *Autoimmunity*.

[42] Marazuela M., García-López M. A., Figueroa-Vega N. (2006). Regulatory T cells in human autoimmune thyroid disease. *The Journal of Clinical Endocrinology & Metabolism*.

[43] Zheng S. G., Wang J. H., Gray J. D., Soucier H., Horwitz D. A. (2004). Natural and induced CD4^+^CD25^+^ cells educate CD4^+^CD25^−^ cells to develop suppressive activity: the role of IL-2, TGF-*β*, and IL-10,. *Journal of Immunology*.

[44] Chistiakov D. A., Turakulov R. I. (2003). CTLA-4 and its role in autoimmune thyroid disease. *Journal of Molecular Endocrinology*.

[45] Nakano A., Watanabe M., Iida T. (2007). Apoptosis-induced decrease of intrathyroidal CD4^+^CD25^+^ regulatory T cells in autoimmune thyroid diseases. *Thyroid*.

[46] Glick A. B., Wodzinski A., Fu P., Levine A. D., Wald D. N. (2013). Impairment of regulatory T-Cell function in autoimmune thyroid disease. *Thyroid*.

[47] Penna G., Roncari A., Amuchastegui S. (2005). Expression of the inhibitory receptor ILT3 on dendritic cells is dispensable for induction of CD4^+^Foxp3^+^ regulatory T cells by 1,25-dihydroxyvitamin D_3_. *Blood*.

[48] Bossowski A., Czarnocka B., Stasiak-Barmuta A., Bardadin K., Urban M., Dadan J. (2007). Analysis of Fas, FasL and Caspase-8 expression in thyroid gland in young patients with immune and non-immune thyroid diseases. *Endokrynologia Polska*.

[49] Łącka K., Maciejewski A. (2012). The role of apoptosis in the etiopathogenesis of autoimmune thyroiditis. *Polski Merkuriusz Lekarski*.

[50] Giordano C., Stassi G., de Maria R. (1997). Potential involvement of fas and its ligand in the pathogenesis of Hashimoto's thyroiditis. *Science*.

[51] Stassi G., Todaro M., Bucchieri F. (1999). Fas/Fas ligand-driven T cell apoptosis as a consequence of ineffective thyroid immunoprivilege in Hashimoto's thyroiditis. *Journal of Immunology*.

[52] Su H. W., Van Antwerp M., Kuick R. (2007). Microarray analysis of cytokine activation of apoptosis pathways in the thyroid. *Endocrinology*.

[53] Baker J. R. (2001). The nature of apoptosis in the thyroid and the role it may play in autoimmune thyroid disease. *Thyroid*.

[54] Fisher G. H., Rosenberg F. J., Straus S. E. (1995). Dominant interfering fas gene mutations impair apoptosis in a human autoimmune lymphoproliferative syndrome. *Cell*.

[55] Stassi G., Zeuner A., di Liberto D., Todaro M., Ricci-Vitiani L., de Maria R. (2001). Fas-FasL in Hashimoto's thyroiditis. *Journal of Clinical Immunology*.

[56] Feldkamp J., Pascher E., Perniok A., Scherbaum W. A. (1999). Fas-mediated apoptosis is inhibited by TSH and iodine in moderate concentrations in primary human thyrocytes in vitro. *Hormone and Metabolic Research*.

[57] Feldkamp J., Pascher E., Schott M., Goretzki P., Seissler J., Scherbaum W. A. (2001). Soluble Fas is increased in hyperthyroidism independent of the underlying thyroid disease. *The Journal of Clinical Endocrinology and Metabolism*.

[58] Bona G., Defranco S., Chiocchetti A. (2003). Defective function of Fas in T cells from paediatric patients with autoimmune thyroid diseases. *Clinical & Experimental Immunology*.

[59] Arata N., Ando T., Unger P., Davies T. F. (2006). By-stander activation in autoimmune thyroiditis: studies on experimental autoimmune thyroiditis in the GFP^+^ fluorescent mouse. *Clinical Immunology*.

[60] Bottazzo G. F., Todd I., Pujol-Borrell R. (1984). Hypotheses on genetic contributions to the aetiology of diabetes mellitus. *Immunology Today*.

[61] Khoury E. L., Pereira L., Greenspan F. S. (1991). Induction of HLA-DR expression on thyroid follicular cells by cytomegalovirus infection in vitro. Evidence for a dual mechanism of induction. *The American Journal of Pathology*.

[62] Neufeld D. S., Platzer M., Davies T. F. (1989). Reovirus induction of MHC class II antigen in rat thyroid cells. *Endocrinology*.

[63] Davies T. F., Walfish P. G., Wall J. R., Volpe R. (1985). The role of human thyroid cell Ia (DR) antigen in thyroid autoimmunity. *Autoimmunity and the Thyroid*.

